# Pediatric Central Nervous System Tumor Overview and Emerging Treatment Considerations

**DOI:** 10.3390/brainsci13071106

**Published:** 2023-07-21

**Authors:** Sudarshawn Damodharan, Diane Puccetti

**Affiliations:** Department of Pediatrics, Division of Pediatric Hematology, Oncology and Bone Marrow Transplant, University of Wisconsin School of Medicine & Public Health, Madison, WI 53792, USA; sudarshawn.damodharan@northwestern.edu

**Keywords:** pediatrics, CNS, tumors, treatment, molecular

## Abstract

Pediatric central nervous system (CNS) tumors are the most common solid tumor in children, with the majority being glial in origin. These tumors are classified by the World Health Organization (WHO) as either being low grade (WHO grade 1 and 2) or high grade (WHO grade 3 and 4). Our knowledge of the molecular landscape of pediatric brain tumors has advanced over the last decade, which has led to newer categorizations along with an expansion of therapeutic targets and options. In this review, we will give an overview of common CNS tumors seen in children along with a focus on treatment options and future considerations.

## 1. Introduction

Pediatric central nervous system (CNS) tumors are the most common solid neoplasm seen in children and are the leading cause of death from cancer in this group [1,2,3]. CNS tumors account for approximately 20% of childhood cancers, second to leukemia in overall frequency [2,4,5]. Recent therapeutic and diagnostic advances have led to improved survival and outcomes for children with brain tumors, but the prognosis for many remains poor. Tumor molecular testing has become more standardized, thus leading to newer classifications as listed by the 5th edition of the World Health Organization (WHO) Classification of CNS tumors [6]. Additionally, this has also altered medical management, with more research and clinical trials targeting these molecular changes. In this review, we will highlight the classifications of common pediatric CNS tumors and review emerging treatment considerations.

## 2. Materials and Methods

This review of common pediatric CNS tumors was written to provide updates on the nomenclature and treatment of these neoplasms focusing on articles published predominantly within the last decade. The 5th edition of the WHO Classification for CNS Tumors (WHO CNS5) was utilized for the classification of the pediatric brain tumors to account for new molecular updates. Clinicaltrials.gov was utilized to find clinical trial identifier numbers.

## 3. Pediatric-Type Diffuse Low-Grade Glioma (LGG)

LGGs are the most common pediatric brain tumor, accounting for approximately one-third of all cases [7,8]. These tumors are designated as WHO grade 1 or 2 and encompass a wide array of histology and varying molecular backgrounds [7]. Many of these tumors are incidentally found on head imaging with intervention needed when adverse symptoms present or when found to have progressive disease. Pediatric diffuse LGGs are primarily heterogenous in nature and can be molecularly classified into distinct subgroups; diffuse astrocytoma MYB- or MYBL1-altered, angiocentric glioma, polymorphous low-grade neuroepithelial tumors, and diffuse LGG MAPK pathway-altered [6].

### 3.1. Diffuse Astrocytoma, MYB- or MYBL1-Altered

This category of pediatric LGG is an infiltrative astroglial neoplasm that is driven by genetic alterations to the MYB or MYBL1 proto-oncogenes [9]. These tumors are typically classified as WHO grade 1. They primarily localize to the cortical and subcortical areas of the brain without further extension [10].

### 3.2. Angiocentric Glioma

These gliomas typically aggregate within the perivascular spaces of the CNS and carry a distinct MYB-QKI gene fusion [11,12]. They are primarily given a WHO grade 1 designation and are most commonly located within the cerebral cortex [11,12].

### 3.3. Polymorphous Low-Grade Neuroepithelial Tumor

These tumors are indolent cerebral neoplasms carrying a WHO grade 1 distinction [13,14]. Alterations within the MAPK pathway play a role in tumor development with most associated with BRAF mutations or FGFR fusions [13]. The primary location of these tumors are within the cortical and subcortical components of the temporal lobes of the brain [15].

### 3.4. Diffuse LGG, MAPK Pathway-Altered

These gliomas occur primarily in children and universally carry an alteration within a gene associated within the MAPK pathway [8]. Typically, they also carry concurrent alterations in FGFR1 or a BRAF fusion [16,17]. They occur throughout the craniospinal axis with some literature pointing to more common alterations depending on the region of occurrence such as a higher incidence of tumors with BRAF fusions within the cerebellum [17]. There is a high prevalence of these tumors in patients with neurofibromatosis type 1 (NF1) with on-going research focusing on targeted therapies to combat this [18,19].

### 3.5. Pilocytic Astrocytoma

Pilocytic astrocytomas are the most common LGG seen in the pediatric population, accounting for approximately 20% of all brain tumors in children and young adults [8,20]. They have a predilection for diseases associated with germline mutations in the MAPK pathway including NF1 and Noonan syndrome [8]. Additional common molecular alterations found include BRAF p.V600E and FGFR1 mutations or fusions [8]. These neoplasms most commonly occur in the cerebellum and are generally slow growing and well circumscribed making them amenable to surgical resection. The majority carry a WHO grade 1 distinction with malignant transformation rarely seen [20].

### 3.6. Treatment

The primary treatment for most LGGs includes an upfront biopsy, if not amenable for a safe gross total resection, to establish the diagnosis and to perform molecular analysis which has now become standard of care [8,21]. This is typically followed by as safe of a gross total resection as possible. Depending on the location of the tumor, resection is sometimes not possible and observation with sequential imaging is carried out as the majority of these tumors remain indolent [21].

In cases where patients with LGGs become clinically symptomatic (i.e., abnormal neurologic symptoms and vision changes) but are unable to have a surgical intervention, treatment with either conventional chemotherapy or radiation therapy has been utilized. In the past, radiation therapy had been used for up-front treatment of LGGs or at the time of progression [22]. A prior phase II study was performed that delivered 54 Gy to the tumor and did result in good progression free survival (PFS) and overall survival rates (OS) of 87% and 96% [23]. However, significant side effects were seen including neurocognitive delays and increased risk of secondary malignancies [23]. Because of these risks, radiation is typically not utilized or offered for LGG management under normal circumstances.

Chemotherapy is utilized more commonly for the medical management of LGGs that cannot be surgically removed. The most commonly utilized systemic chemotherapy regimens for LGG in children include carboplatin and vincristine or vinblastine alone [7]. These chemotherapy regimens have led to similar PFS to those who received radiation therapy but with significantly reduced toxicity and side effect profiles in multiple phase II studies performed in children with LGGs [24,25].

Given the increased molecular understanding of pediatric LGGs, more targeted agents have become available primarily targeting the MAPK pathway [8] (Table 1). The two most common agents being utilized are MEK and BRAF inhibitors. Selumetinib is a selective small molecule inhibitor of MEK-1/2 which has shown promising results in this setting. Most recently, a phase II trial was conducted for selumetinib for pediatric LGG in patients without NF1 via the Pediatric Brain Tumor Consortium (PBTC) [26]. This showed either stable to improved disease status in patients with either recurrent or progressive LGGs with further studies on-going [26]. Currently, the Children’s Oncology Group (COG) is conducting a phase III randomized clinical trial assessing upfront selumetinib to standard chemotherapy (carboplatin and vincristine) among patients with and without NF1 associated LGGs (NCT04166409). Dabrafenib and vemurafenib are small molecule BRAF kinase inhibitors that have also been utilized in LGGs that harbor BRAF V600E mutations. A phase I trial conducted by the Pacific Neuro-Oncology Consortium (PNOC) looked to assess the utility of vemurafenib in children with recurrent or refractory gliomas harboring the BRAF V600E mutation [27]. The trial showed that approximately half the patients had a positive response to vemurafenib and that the drug was well tolerated [27]. More on-going therapies are looking at MAPK combination therapies with both MEK and BRAF inhibitors with preliminary results from an on-going phase II study showing improved response rates and PFS to the agents individually or to standard chemotherapy (NCT02684058). A phase 2 study evaluating the pan-RAF inhibitor tovorafenib (DAY101) has also shown promising preliminary results for refractory LGGs in children with BRAF alterations (NCT04775485). There is still much to be learned about molecular inhibitors, with long-term toxicities not known, but they do pose a more targeted approach for the medical management of pediatric LGG.

## 4. Pediatric-Type Diffuse High-Grade Gliomas (HGG)

HGGs account for approximately 10% of all pediatric CNS tumors and carry a poor prognosis [28]. Despite surgical and medical interventions, these tumors remain fatal, with the majority of children succumbing to their disease within two years of diagnosis [29]. The primary subtypes of pediatric HGGs includes diffuse midline glioma (DMG) H3 K27-altered, diffuse HGG H3-wildtype (WT) and IDH-WT, diffuse hemispheric glioma H3 G34-mutant, and infant-type hemispheric glioma [6].

### 4.1. DMG, H3 K27-Altered

These gliomas are extremely infiltrative neoplasms primarily carrying a missense mutation at histone H3 with a lysine to methionine substitution at point 27 (K27M) [30]. The mutation primarily take place at either *H3F3A* or *HIST1H3B*, with those with an alteration at *H3F3A* having a more severe phenotype [31]. Additional subtypes of this tumor include H3-wildtype (WT) with EZHIP overexpression and EGFR-mutant [32]. These tumors carry a WHO grade 4 distinction and are universally fatal. The preferential location for these neoplasms is within the brainstem or pons but may also present in the thalamus or spinal cord [33,34]. The survival for these patients is poor, with the majority succumbing to their disease within a year of diagnosis [35].

### 4.2. Diffuse Pediatric HGG, H3-WT and IDH-WT

These diffuse gliomas carry similar clinical characteristics as H3 K27-altered DMG but do not possess the typical mutations associated with those neoplasms. As such, they also carry a WHO grade 4 designation and are universally fatal. These tumors tend to have alterations within the receptor tyrosine kinase (RTK) pathway with a smaller subgroup also having upregulation of MYCN [36,37].

### 4.3. Diffuse Hemispheric Glioma (DHG), H3 G34-Mutant

DHG is an aggressive malignancy that involves the cerebral hemispheres, carrying a WHO grade 4 classification. They are characterized by a missense mutation of the H3-3A gene, primarily resulting in substitution of glycine to arginine at point 34 (G34) in histone H3 [38]. There is also upregulation of the MYCN protooncogene that coexists with the H3 G34 mutation in the majority of these tumors [39]. DHG primarily affects young adults with a very minor population in young children [38]. 

### 4.4. Infant-Type Hemispheric Glioma

This neoplasm is a highly cellular astrocytoma that occurs very early on in childhood with a median age of 3 months [40,41] They typically involve RTK fusions with the most common being within the NTRK, ROS1, MET or ALK pathways [40,41]. These tumors almost universally present in the supratentorial CNS area and are typically amenable to surgical resection along with targeted medical intervention. Given this, the survival for these patients is improved compared to other pediatric HGGs.

### 4.5. Treatment

An initial biopsy or subtotal resection is performed to establish the diagnosis of a pediatric HGG along with pursuing further molecular characterization. Surgical intervention then follows this, however most pediatric HGGs are not amenable to a gross resection. Currently, there are no curative treatments for DMGs and many other pediatric HGGs. Additionally, these tumors are extremely chemo-resistant and hard to target due to the prohibitive nature of the blood–brain barrier (BBB) [42,43]. Current standard treatment includes radiation, extending OS by months at most for patients with a DMG [44,45]. Many newer therapeutic options have emerged including targeted small molecule inhibitors along with immunotherapeutic considerations to combat these fatal neoplasms.

Various targeted therapies are currently under investigation for the treatment of pediatric HGGs with much of the on-going research being performed on H3 K27-altered DMG. Panobinostat is a histone deacetylase (HDAC) inhibitor which has shown preclinical efficacy to normalize mutant K27M expression and reduce tumor proliferation in preclinical murine models [46,47]. A phase I trial of panobinostat following radiation therapy was initiated by the PBTC with preliminary results unfortunately not showing significant improvement in PFS or OS in patients with H3 K27-altered gliomas (NCT02717455). This is thought to be contributed by the fact that in humans, it may not cross the BBB as shown in murine models [48]. ONC201 is another targeted agent and is an antagonist against dopamine receptors DRD2/3 and has shown efficacy in targeting various cancers including CNS malignancies [49,50]. An ongoing phase III trial is looking to assess ONC201 for treatment against H3 K27-altered DMG (NCT05580562). Larotrectinib has also been utilized effectively in TRK-fusion positive CNS tumors showing durable responses with a favorable safety profile [41]. Further research is on-going to find targeted agents against pediatric HGGs as we learn more about the molecular characterization of these neoplasms.

Immunotherapy is an evolving field that utilizes the body’s immune system to target and get rid of cancer cells. Many on-going investigations are looking to utilize this technology to combat pediatric HGGs (Table 2). This therapy includes immunovirotherapy, checkpoint blockade and chimeric antigen receptor (CAR) T cell therapy. Immune checkpoint receptors and ligands are expressed on activated T cells with checkpoint blockade looking to target these to prevent inactivation and elicit on-going anti-tumor effects [51]. Various monoclonal antibodies (mAb) have been developed primarily targeting protein PD-1 and ligand PD-L1 which play crucial roles in checkpoint blockade [52]. These mAbs have shown promising results in adult cancers but have been less studied in pediatric malignancies. Currently, an on-going phase I clinical trial is assessing the combination of checkpoint blockade in combination with a peptide vaccine for H3 K27-altered diffuse midline gliomas (NCT04943848). Additionally, a phase I trial with checkpoint blockade monotherapy was conducted for patients with pediatric high-grade gliomas (NCT02359565) with preliminary results for their DMG cohort unfortunately showing a worse PFS compared to the historical median. Checkpoint blockade’s effect on pediatric CNS tumors still remains unknown with further research needed to continue to investigate this. CAR-T cell therapy has also been a primary focus with most of the newer therapies and research looking to utilize this for H3 K27-altered DMGs. There had been two phase I CAR-T cell clinical trials targeting B7H3 and GD2 (NCT04185038 & NCT04196413) with more currently underway. Preliminary results from the GD2 CAR-T cell trial have shown promising results with three of four initial patients showing significant clinical and radiographic improvements [53]. Immunovirotherapy and vaccine therapies are also a consideration with on-going trials looking to assess the efficacy in pediatric HGGs (NCT04943848, NCT03396575, NCT04978727). Immunotherapeutic options against pediatric high-grade gliomas continues to be a focal point of many research endeavors with more promising options on the horizon.

## 5. Ependymal Tumors

**Figure 1:**
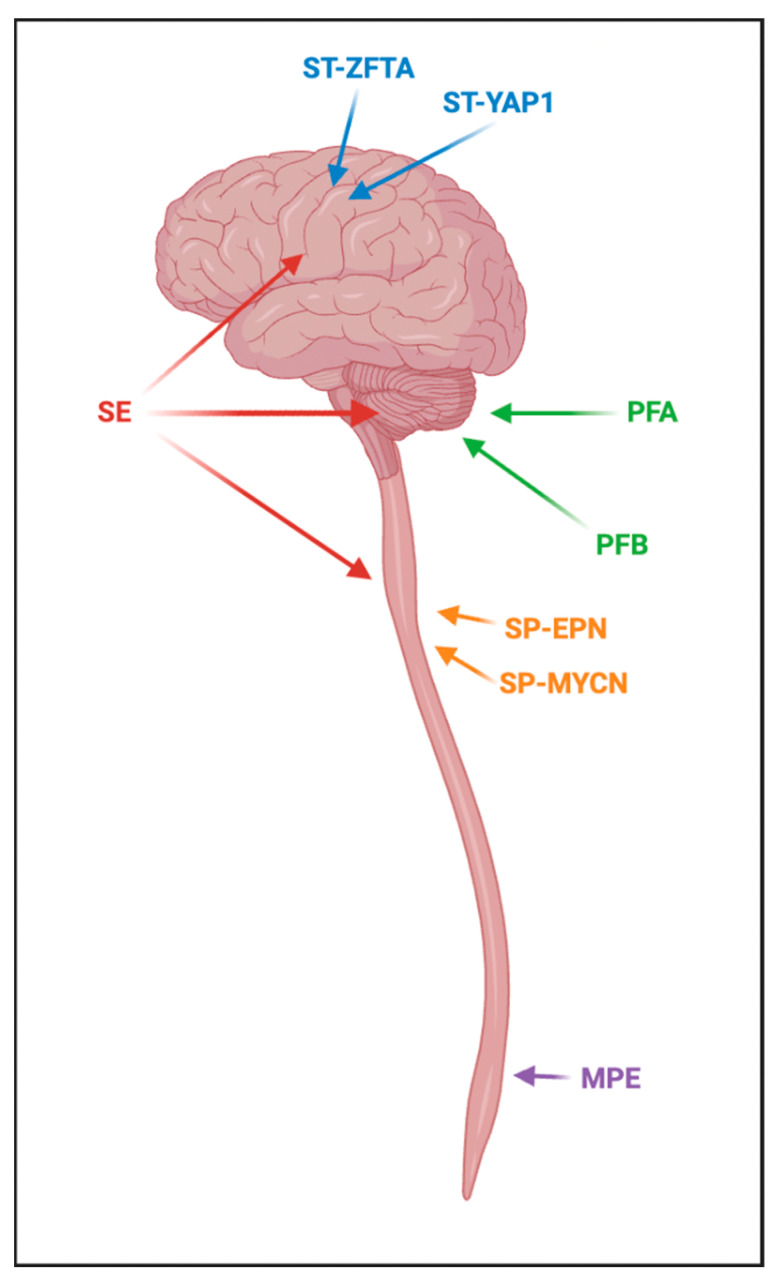
Ependymoma. Here, we show the differing subtypes of ependymoma along with their commonly found anatomical locations within the central nervous system (CNS). ST-ZFTA and ST-YAP1 are typically located in the supratentorial compartment of the CNS. SE are typically indolent and do not have a predilection for one compartment and occur in all three–supratentorial, posterior fossa, and spinal. PFA and PFB occur infratentorially within the posterior fossa. SP-EPN and SP-MYCN occur most commonly within the cervical and thoracic portions of the spinal cord. Finally, MPE are slow-growing tumors that occur most commonly in the lower spinal region. SE—subependymoma; ST-ZFTA—supratentorial, ZFTA-fusion-positive ependymoma; ST-YAP1—supratentorial, YAP1-fusion-positive ependymoma; PFA—group A posterior fossa ependymoma; PFB—group B posterior fossa ependymoma; SP-EPN—spinal ependymoma; SP-MYCN—spinal ependymoma, MYCN-amplified; MPE—myxopapillary ependymoma.

Ependymomas are the third most common CNS tumor in children, accounting for approximately 5–10% of these neoplasms and are a subtype of gliomas [54]. These tumors are classified utilizing a combination of histologic, molecular and anatomical characteristics. There are currently eight subtypes of these tumors defined per the WHO CNS 5 (Figure 1) [6]. The mainstay of therapy is surgical resection followed by adjuvant radiation [54]. Here, we will expand on four of the distinct subtypes of pediatric ependymomas; supratentorial ependymoma ZFTA fusion-positive, supratentorial ependymoma YAP1 fusion-positive, posterior fossa group A (PFA) ependymoma, and posterior fossa group B (PFB) ependymoma.

### 5.1. Supratentorial Ependymoma, ZFTA Fusion-Positive

These neoplasms are characterized by a ZFTA fusion gene, with the majority fused with RELA as the primary oncogenic driver [55]. They primarily arise in the frontal or parietal lobes, displaying varied anaplasia and are typically categorized as either a WHO grade 2 or 3. In a prior retrospective analysis including both children and adults, lower survival rates were seen in ZFTA fusion-positive ependymomas compared to other subtypes in this location [56]. However, newer prospective pediatric literature suggests this to not be true with further outcomes research on-going [57].

### 5.2. Supratentorial Ependymoma, YAP1 Fusion-Positive

This subtype of ependymomas carry a typical YAP1 fusion gene primarily fused with MAMLD1 [54]. These tumors are also primarily graded as WHO 2 or 3. The YAP1 fusion gene seems to be restricted to younger children with most patients presenting at a young age at the time of diagnosis [54,58]. In a large retrospective analysis, children with YAP1 fusion-positive ependymomas had more favorable outcomes than those of other supratentorial ependymoma subtypes [57].

### 5.3. PFA Ependymoma

These tumors occur within the posterior fossa of the brain and are classified by a loss of nuclear histone H3 K27 trimethylation in tumor cells along with global DNA hypomethylation [59]. The majority of these tumors also carry a gain of chromosome 1q but this does not seem to have any prognostic significance [60]. They are typically graded as WHO 2 or 3 based upon the degree of anaplasia present but tend to have poorer outcomes with on-going studies looking to further explore the reason for this [61]. PFA ependymomas primarily occur in infants and young children [54].

### 5.4. PFB Ependymoma

Unlike PFA, PFB ependymomas are classified by retention of nuclear H3 K27M trimethylation [54]. The most common chromosomal aberrations associated with these neoplasms include loss of 22q, monosomy 6 and trisomy 18 [62]. They occur more commonly in young adults and less commonly in children [54]. PFB ependymomas have generally carried a favorable prognosis with newer research looking into decreasing the amount of adjuvant radiation delivered after surgery [61].

### 5.5. Treatment

The diagnosis of an ependymoma is confirmed via an upfront resection or after biopsy via pathology and molecular characterization. The primary management involves as complete of a surgical resection along with adjuvant radiation therapy. Prior studies have shown the extent of surgical resection to be the most significant prognostic factor for children with ependymomas [57,63,64]. Adjuvant radiation therapy has remained the mainstay consolidative approach with the role for chemotherapy continuing to be studied. Optimizing the dose of adjuvant radiation therapy along with studying the use of targeted inhibitors have been the focus of on-going research.

Radiation therapy has remained a crucial component of ependymoma therapy. Multiple prospective studies have shown the effectiveness of consolidative radiation therapy for pediatric ependymomas regardless of anatomical location with increases in both PFS and OS [65,66]. Additionally, Massimino et al. advocated for local intensification with an additional boost of radiation therapy to the primary tumor bed given the favorable outcomes seen after this [65]. The age to consider radiation therapy for children with ependymomas remains an on-going debate with recent literature suggesting as early as 12 months [57,67]. Further studies are needed to continue to assess the long-term neurocognitive outcomes of these patients’ following radiation. Newer investigations are looking into an observation only approach for WHO grade 2 ependymomas that achieve a gross total resection (NCT01096368 and NCT02265770). Additionally, a phase III COG phase study prospectively randomized patients to either receive radiation therapy alone versus radiation and maintenance chemotherapy for children with supratentorial and PFB ependymomas [67]. Results from this trial suggests comparable 3-year event free survival (EFS) in both cohorts but that some populations may benefit from maintenance chemotherapy [67]. Further investigations to optimize adjuvant therapy for pediatric ependymomas remains on-going.

Newer molecular characterization of pediatric ependymomas have also led to continued efforts to find targeted agents to combat these neoplasms. Supratentorial ependymomas with a CDKN2A deletion have been found in multiple studies to have a more aggressive phenotype and it is thought that therapy for these tumors should be intensified [68,69]. Palbociclib is an oral small molecule inhibitor of CDK4/CDK6 and is currently being studied in children with CNS tumors with amplification of this pathway including ependymomas [70]. Further research is looking to better understand the molecular landscape of pediatric ependymomas to tailor future targeted treatments

## 6. CNS Embryonal Tumors

Embryonal tumors account for approximately 20% of all pediatric brain tumors, with a typical histology consisting of small, round, blue cells [71]. Previously, these tumors were categorized as primitive neuroepithelial tumors given their cell of origin [71]. Newer molecular data have led to the reclassification of these tumors based upon a combination of their oncogenic drivers along with histology. Medulloblastoma is the primary embryonal tumor that occurs in children with the WHO CNS 5 listing all others under the category as “other CNS embryonal tumors” [6]. Tumors included within this are atypical teratoid/rhabdoid tumor (AT/RT), cribriform neuroepithelial tumors and CNS neuroblastoma FOXR2-activated [6]. We will expand further on medulloblastoma given its increased incidence.

### 6.1. Medulloblastoma

**Figure 2:**
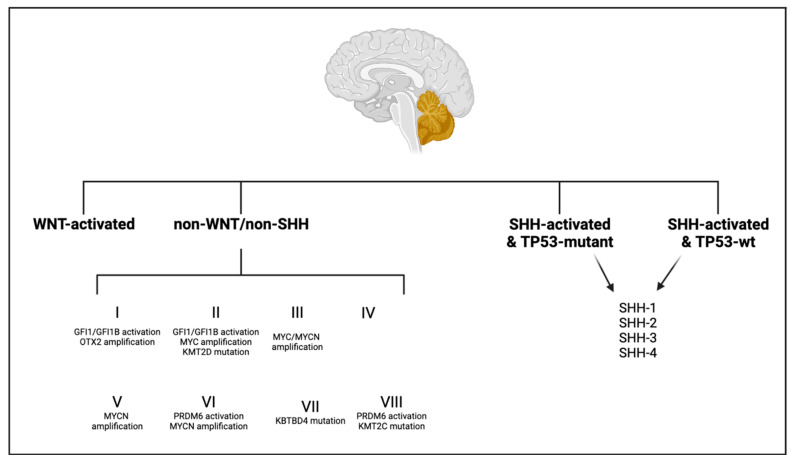
Medulloblastoma. Medulloblastoma is grouped into 4 distinct molecular subtypes–WNT-activated, non-WNT/non-SHH, SHH-activated and TP53-mutant, SHH-activated and TP53-wildtype (WT). WNT-activated medulloblastoma have the most favorable prognosis of all the subtypes. SHH-activated medulloblastoma can be further broken down to four provisional subclassifications–SHH-1, SHH-2, SHH-3 and SHH-4. On-going work is looking to define the specific molecular and cytogenetic aberrations within these subclassifications of SHH-activated medulloblastoma. Those that occur with a with a TP53 mutation tend to have germline alterations and are more commonly found in cancer predisposing syndromes. Non-WNT/non-SHH medulloblastoma encompass the prior classification of group 3 and group 4 and have the worst prognosis, showing high resistance to conventional treatment. Northcott et al. further defined this subgroup as having eight distinct subclassifications (I–VIII) with their key molecular abnormalities listed within the figure [83,84]. Still, there is much to be learned about this subtype of medulloblastoma to improve current treatments.

Medulloblastoma is the most common malignant pediatric brain tumor, typically arising from the cerebellum [72]. It can arise at all ages but most commonly occurs in childhood, accounting for approximately 20% of all CNS neoplasms in this population [73]. All medulloblastoma are considered WHO grade 4 even though some of its subgroups carry a more favorable prognosis than others. Initially, medulloblastoma was classified as being WNT-activated, SHH-activated, group 3 and group 4. With newer advances in its molecular landscape, medulloblastoma is now classified into four distinct molecular subtypes; WNT-activated, SHH-activated and TP53-WT, SHH-activated and TP53-mutant, and non-WNT/non-SHH which primarily comprise group 3 and group 4 types of this neoplasm [74]. In total, there are four subgroups of SHH medulloblastoma and eight subgroups of non-WNT/non-SHH (group 3/4) medulloblastoma (Figure 2). We will expand further upon these distinct molecular subtypes of pediatric medulloblastoma.

#### 6.1.1. Medulloblastoma, WNT-Activated

These neoplasms arise primarily from the posterior brainstem or cerebellum with characteristic activation of the WNT signaling pathway [75]. These account for approximately 10% of all medulloblastomas and occur primarily in middle aged children with the median age of diagnosis being 10 years of age [76]. These neoplasms carry a more favorable prognosis compared to the other subtypes of medulloblastoma with 5-year survival rates approaching nearly 100% in children with current surgical and medical interventions [77].

#### 6.1.2. Medulloblastoma, SHH-Activated and TP53-WT

These tumors demonstrate activation of the SHH signaling pathway along with a WT TP53 gene. SHH-activated medulloblastoma have four molecular subgroups SHH-1, SHH-2, SHH-3, and SHH-4 [78]. Overall prognosis for these patients depends on staging at the time diagnosis (Table 3) along with assessing for MYCN amplification, which is a poor prognostic factor [79]. In the absence of these high-risk features, outcomes are favorable with survival nearing 80% [77].

#### 6.1.3. Medulloblastoma, SHH-Activated and TP53-Mutant

This molecular subtype of medulloblastoma shows activation of the SHH pathway and mutant-TP53. Concurrent TP53 mutation, MYCN amplification is present in approximately 10–15% of all pediatric SHH-activated medulloblastoma [77]. These tumors typically present with high-risk features including an increased incidence of leptomeningeal involvement at the time of diagnosis [80]. There is an increased incidence of poor outcomes with this subtype of pediatric medulloblastoma which is thought to be related to resistance to conventional post-surgical adjuvant treatment [81].

#### 6.1.4. Medulloblastoma, Non-WNT/Non-SHH

These tumors are classified as group 3 and group 4 medulloblastoma and are comprised of eight molecular subgroups (Figure 2). Overexpression of MYC is commonly associated with group 3 medulloblastoma, occurring in almost a fifth of these neoplasms with MYCN accounting for approximately 5% of this [82]. Abnormalities in chromosome 17 occur in the majority of group 3 and 4 medulloblastoma, as well [83]. This class of medulloblastoma portrays the worst prognosis which is thought to be related to the high incidence of MYCN amplification. Further molecular characterization is needed for this subtype of medulloblastoma to assess changes that may have prognostic and therapeutic significance.

#### 6.1.5. Treatment

The standard treatment for pediatric medulloblastoma consists of a multimodality approach with surgery to complete as total of a resection as able along with adjuvant radiation therapy and systemic chemotherapy. Newer on-going investigations are utilizing molecular characteristics to risk stratify patients to receive differing amounts of radiation and chemotherapy to minimize adverse symptoms and neurocognitive decline. Targeted therapies also continue to be researched including small molecule inhibitors and immunotherapeutic options to combat these neoplasms.

Currently, for children over the age of 3 postoperative craniospinal (CSI) radiation is the standard of care with an additional boost given to the primary tumor bed [85]. This is usually combined with concurrent radio-sensitizing chemotherapy and followed with further maintenance chemotherapy. The Head Start IV randomized clinical trial offered by the National Experimental Therapeutics Consortium (NEXT) is looking to assess a radiation sparing approach to treat children with medulloblastoma who are 10 years or younger (NCT02875314). Instead, patients enrolled on this treatment protocol receive more intensive chemotherapy including high-dose chemotherapy with stem-cell rescue. Results from the prior Head Start III trial showed promising results for children with certain subtypes of medulloblastoma with most patient surviving without irradiation [86]. St. Jude also has an on-going clinical trial to risk stratify patients to receive differing doses radiation based upon the molecular profile of the tumor (NCT01878617). Research is on-going to assess the dose of adjuvant radiation needed based upon molecular characterization and risk stratification to limit long-term side effects.

Research efforts are looking to find targeted therapies and immunotherapeutic options for medulloblastoma. Vismodegib is a small molecule inhibitor of the SHH pathway which has shown some efficacy against SHH-activated medulloblastoma in adults [87]. An on-going clinical trial at St. Jude is investing this in combination with adjuvant chemotherapy for SHH-activated medulloblastoma in children (NCT01878617). Another on-going phase I/II study out of the PBTC is assessing silmitasertib in children with recurrent or refractory SHH-activated medulloblastoma (NCT03904862). Silmitasertib is a CK2 inhibitor which inhibits transcription factor GLI which is a terminal effector within the SHH pathway with hopes that this may prevent tumor proliferation [88]. Driver mutations and inhibitors that play a role in tumor proliferation have been identified in WNT and SHH altered medulloblastoma with further characterization needed to better understand group 3 and 4 tumors that tend to have the worst outcomes. Immunotherapy is another avenue of on-going treatment for pediatric medulloblastoma. HER2 has been shown to be overexpressed on the surface of medulloblastoma with on-going efforts to assess the utility of an anti-HER2 CAR-T cells for these tumors [89]. This has proven to be effective in various preclinical murine models [89,90]. There is an on-going phase I study looking at the utility of HER2-specific CAR-T cell therapy for pediatric CNS tumors (NCT03500991). Antigen escape is a worry given tumor heterogeneity with on-going investigations looking to target multiple surface antigen at the same time [91].

Clinical staging includes magnetic resonance imaging of the CNS and lumbar puncture to assess cerebrospinal fluid cytology. This staging system to assess the metastatic spread of medulloblastoma was developed by Chang et al. in 1969 and is still used today [92].

## 7. Conclusions

Advances in sequencing have allowed for newer molecular categorizations of pediatric brain tumors per the latest WHO CNS classification system. Continued rapid advances in the molecular understanding and treatments for these neoplasms are on-going. Though the prognosis for many of these tumors remains poor, newer targeted therapies and medical advances remain on the horizon, with the hope that these will improve outcomes.

## Figures and Tables

**Table 1:** Active clinical trials for pediatric LGG utilizing targeted therapies.

Study Title	NCT Number	Targeted Therapeutic Intervention	Country
DAY101 vs. Standard of Care Chemotherapy in Pediatric Patients with Low-Grade Glioma Requiring First-Line Systemic Therapy (LOGGIC/FIREFLY-2)	NCT05566795	Drug: DAY101	USA, Canada, Czechia, Korea, Switzerland
A Study to Evaluate DAY101 in Pediatric and Young Adult Patients with Relapsed or Progressive Low-Grade Glioma and Advance Solid Tumors	NCT04775485	Drug: DAY101	USA
SJ901: Evaluation of Mirdametinib in Children, Adolescents, and Young Adults with Low-Grade Glioma	NCT04923126	Drug: Mirdametinib	USA
A Study of the Drugs Selumetinib Versus Carboplatin/ Vincristine in Patients with Neurofibromatosis and Low-Grade Glioma	NCT03871257	Drug: Selumetinib Sulfate	USA, Canada
A Study of the Drugs Selumetinib vs. Carboplatin and Vincristine in Patients with Low-Grade Glioma	NCT04166409	Drug: Selumetinib Sulfate	USA, Canada
A Study to Compare Treatment with the Drug Selumetinib Alone Versus Selumetinib and Vinblastine in Patients with Recurrent or Progressive Low-Grade Glioma	NCT04576117	Drug: Selumetinib Sulfate	USA
Trametinib and Everolimus for Treatment of Pediatric and Young Adult Patients with Recurrent Gliomas	NCT04485559	Drug: Everolimus Drug: Trametinib	USA
A Trial of Dabrafenib, Trametinib and Hydroxychloroquine for Patients with Recurrent LGG or HGG With a BRAF Aberration	NCT04201457	Drug: Dabrafenib Drug: Trametinib	USA
Pediatric Low-Grade Glioma–MEK inhibitor Trial vs Chemotherapy	NCT05180825	Drug: Trametinib	France
BGB-290 and Temozolomide in Treating Isocitrate Dehydrogenase (IDH)1/2-Mutant Grade I-IV Gliomas	NCT03749187	Drug: PARP Inhibitor BGB-290	USA
Pediatric Long-Term Follow-up and Rollover Study	NCT03975829	Drug: Dabrafenib Drug: Trametinib	USA

Clinical trials found on www.clinicaltrials.gov as of 5 June 2023.

**Table 2:** Active clinical trials for pediatric HGG utilizing immunotherapy.

Study Title	NCT Number	Immunotherapy Intervention	Country
Nivolumab in Combination with Temozolomide and Radiotherapy in Childrenand Adolescents with Newly Diagnosed High-grade Glioma	NCT04267146	Drug: Nivolumab	France
Pembrolizumab in Treating Younger Patients with Recurrent, Progressive, or Refractory High-Grade Gliomas, Diffuse Intrinsic Pontine Gliomas, Hypermutated Brain Tumors, Ependymoma or Medulloblastoma	NCT02359565	Drug: Pembrolizumab	USA, Canada
A Pilot Study of SurVaxMin Children Progressive or Relapsed Medulloblastoma, High Grade Glioma, Ependymoma and Newly Diagnosed Diffuse Intrinsic Pontine Glioma	NCT04978727	Drug: SurVaxM	USA
REGN2810 in Pediatric Patients with Relapsed, Refractory Solid, or Central Nervous System (CNS) Tumors and Safety and Efficacy of REGN2810 in Combination with Radiotherapy in Pediatric Patients with Newly Diagnosed or Recurrent Glioma	NCT03690869	Drug: Cemiplimab	USA
Autologous Dendritic Cells, Metronomic Cyclophosphamide and Checkpoint Blockade in Children with Relapsed HGG	NCT03879512	Drug: Cancer vaccine and checkpoint blockade	Germany
C7R-GD2.CAR T Cells for Patients with GD2-expressing Brain Tumors (GAIL-B)	NCT04099797	Drug: GD2-CART cells	USA
Loc3CAR: Locoregional Delivery of B7-H3-CAR T Cells for Pediatric Patients with Primary CNS Tumors	NCT05835687	Drug: B7-H3 CART cells	USA
rHSC-DIPGVax Plus Checkpoint Blockade for the Treatment of Newly Diagnosed DIPG and DMG	NCT04943848	Drug: rHSC- DIPGVax Drug: Balstilimab Drug: Zalifrelimab	USA

Clinical trials found on www.clinicaltrials.gov as of 5 June 2023.

**Table 3:** Medulloblastoma staging.

Stage	Extent of Disease
M0	No evidence of subarachnoid or hematogenous metastasis
M1	Microscopic tumor cells found in the CSF
M2	Gross nodular seeding demonstrated in the cerebellar/cerebral subarachnoid space or in the third or lateral ventricles
M3	Gross nodular seeding in the spinal subarachnoid space
M4	Metastasis outside the cerebrospinal axis

## Data Availability

Data sharing not applicable.
